# Process-Specific Blood Biomarkers and Outcomes in COVID-19 Versus Non-COVID-19 ARDS (APEL–COVID Study): A Prospective, Observational Cohort Study

**DOI:** 10.3390/jcm13195919

**Published:** 2024-10-04

**Authors:** Olivier Lesur, Eric David Segal, Kevin Rego, Alain Mercat, Pierre Asfar, Frédéric Chagnon

**Affiliations:** 1 Centre de Recherche Clinique du CHU Sherbrooke (CRCHUS), Department of Intensive Care Medicine, Faculty of Medicine and Health Sciences, University of Sherbrooke, 3001 12th Avenue Nord, Sherbrooke, QC J1H 5N4, Canada; kevin.rego@usherbrooke.ca; 2 Départements de Soins Intensifs et Service de Pneumologie, CHU Sherbrooke, 3001, 12th Avenue Nord, Sherbrooke QC J1H 5N4, Canada; 3 Département de Médecine, CHU Sherbrooke, Faculté de Médecine et des Sciences de la Santé, Université de Sherbrooke, Sherbrooke, QC J1H 5N4, Canada; eric.segal.chum@ssss.gouv.qc.ca; 4 Département de Médecine Intensive-Réanimation, CHU Angers, 49000 Angers, France; almercat@chu-angers.fr (A.M.); piasfar@chu-angers.fr (P.A.)

**Keywords:** COVID-19, SARS, ARDS, apelins (APL), NEP, ACE2, LOX

## Abstract

**Background:** Severe acute respiratory syndrome (SARS) and acute respiratory distress syndrome (ARDS) are often considered separate clinico-radiological entities. Whether these conditions also present a single process-specific systemic biomolecular phenotype and how this relates to patient outcomes remains unknown. A prospective cohort study was conducted, including adult patients admitted to the ICU and general floors for COVID-19-related (COVID+) or non-COVID-19-related (COVID−) acute respiratory failure during the main phase of the pandemic. The primary objective was to study blood biomarkers and outcomes among different groups and severity subsets. **Results:** A total of 132 patients were included, as follows: 67 COVID+, 54 COVID− (with 11 matched control subjects for biomarker reference), and 58 of these patients allowed for further pre- and post-analysis. The baseline apelin (APL) levels were higher in COVID+ patients (*p* < 0.0001 vs. COVID− patients) and in SARS COVID+ patients (*p* ≤ 0.02 vs. ARDS), while the IL-6 levels were higher in ARDS COVID− patients (*p* ≤ 0.0001 vs. SARS). Multivariable logistic regression analyses with cohort biomarkers and outcome parameters revealed the following: (i) log-transformed neprilysin (NEP) activity was significantly higher in COVID+ patients (1.11 [95% CI: 0.4–1.9] vs. 0.37 [95% CI: 0.1–0.8], fold change (FC): 1.43 [95% CI: 1.04–1.97], *p* = 0.029) and in SARS patients (FC: 1.65 [95% CI: 1.05–2.6], *p* = 0.032 vs. non-SARS COVID+ patients, and 1.73 [95% CI: 1.19–2.5], *p* = 0.005 vs. ARDS COVID− patients) and (ii) higher lysyl oxidase (LOX) activity and APL levels were respectively associated with death and a shorter length of hospital stay in SARS COVID+ patients (Odds Ratios (OR): 1.01 [1.00–1.02], *p* = 0.05, and OR: −0.007 [−0.013–0.0001], *p* = 0.048). **Conclusion:** Process-specific blood biomarkers exhibited distinct profiles between COVID+ and COVID− patients, and across stages of severity. NEP and LOX activities, as well as APL levels, are particularly linked to COVID+ patients and their outcomes (ClinicalTrials.gov Identifier: NCT04632732).

## 1. Introduction

The lungs were a primary target during the COVID-19 pandemic, and the pooled prevalence of acute respiratory distress syndrome (ARDS) in symptomatic adult patients has recently been estimated to exceed 30% worldwide [1]. This severe form, specifically referred to as coronavirus-related severe acute respiratory syndrome 2 (SARS-CoV-2, subsequently called SARS), exhibits major alterations in the oxygenation index [2]. This coronavirus, like others, enters the respiratory system via microdroplets aspirated or ingested from contaminated hands or objects, subsequently integrating into cellular machinery to replicate. SARS develops within approximately two weeks of the symptom onset, often with recurrent high-grade fever, and has a mortality rate ranging from 10% to 50% [3]. SARS is considered a sub-phenotype or endotype of traditional ARDS, with specific trends and variations [4,5].

In SARS, pulmonary infiltrates are often indicative of hyperinflammatory airspaces [6], with alveolar–capillary barrier (ACB) hyperpermeability [7,8,9]. The combined circulatory and parenchymal injuries result in exceptionally severe hypoxemia [5], and issues related to lung tissue remodeling, repair, and fibrosis are highly prevalent [10,11]. These events are central to acute lung injury (ALI) and its repair [8,12] and can be tentatively assessed using specific biomarkers. Due to the specificities mentioned, a distinctive biomarker profile can be anticipated between SARS and ARDS.

Interleukin-6 (IL-6), as a mediator of the acute-phase inflammatory response in sepsis, ARDS, and COVID-19, is a key player in the ‘cytokine storm’ often observed and associated with ACB hyperpermeability [13,14]. Surfactant protein D (SP-D) is a specific protein produced by type II alveolar epithelial cells (AECII) in the lungs, released into the bloodstream when the alveolar–capillary barrier (ACB) becomes hyperpermeable due to ALI or aggressive cyclic stretching and/or mechanical ventilation. SP-D serves as a diagnostic and prognostic biosensor for ACB bloodstream leakage in ARDS and lung fibrosis [15,16,17]. Lysyl oxidase (LOX) is an important enzyme that stabilizes the extracellular protein network of collagen and elastin during tissue remodeling. Enhanced LOX expression or activity is a valuable marker of lung fibrosis progression [18,19,20,21].

Recent research has highlighted the apelin (APL, ≠isoforms)/APJ (apelin receptor) system as a key player in cardiovascular homeostasis [22,23], as well as a novel regulator of lung tissue protection and repair. This system stabilizes mitochondrial function, reduces ACB permeability on both sides, and supports microvascular regeneration by suppressing transforming growth factor beta (TGFβ)-induced endothelial-to-mesenchymal transition [24,25,26,27,28,29,30,31]. APJ is known as a co-receptor for immunodeficiency viruses [32], and its entry is blocked by APL [33]. Meanwhile, angiotensin-converting enzyme 2 (ACE2) and neprilysin (NEP) are degrading enzymes for APL in the renin–angiotensin system (RAS) [30,31]. ACE2 and APJ are located close to each other within lung AECII membranes [33,34]. In the cardiovascular system, ACE2 is a promoter of vasodilation; anti-inflammation; and tissue protection. In the lungs, ACE2 is abundantly expressed in AECII and serves as an entry point for viruses, such as CoV-1 and -2. In this respect, APL inhibits cell-to-cell fusion mediated by ACE2 binding to CoV-2 surface S proteins [35,36,37]. NEP is an integral membrane-bound endopeptidase that is widely distributed and plays a crucial role in the degradation and turnover of vasoactive peptides, as well as in the regulation of tissue inflammation [31]. NEP activity in alveolar airspaces has been reported to be elevated in experimental and clinical ALI/ARDS, and NEP has been proposed as a target for a therapeutic approach to COVID-19 [38,39].

**Hypothesis 1.**  *A dimorphic, process-specific biomarker signature can be observed between COVID-19 and non-COVID-19 respiratory injuries, with some aspects associated with outcomes.*

OBJECTIVES:

Primary:

1. To establish the involvement of APL and related systems (RAS and APL-degrading enzymes ACE2 and NEP), and to assess the dominance of lung inflammation, hyperpermeability, and subsequent remodeling/fibrosis (LOX activity) in severe COVID-19 forms (hereafter referred to as COVID+ SARS);
2. To compare this profile with ARDS related to non-COVID-19 causes (hereafter referred to as COVID−);

Secondary: To establish links with baseline and evolving clinical data.

## 2. Study Design, Materials, and Methods

### 2.1. Study Design

This was an observational, prospective cohort study conducted at two sites (Sherbrooke, QC, Canada, and Angers, France), with an additional control subject group used as a gold standard reference for biomarker comparisons. The study adhered to the STROBE guidelines for cohort studies and was officially registered with ClinicalTrials.gov (Identifier: NCT04632732). The inclusion period ran from the end of October 2020 to mid-September 2021.

### 2.2. Patient Screening and Sample Calculation

All patients admitted to the Intensive Care Units at both sites were screened by the respective research staff to assess their eligibility. Patient information was recorded in a screening log (not done in the 2nd site, including 15% of the patient’s cohort). Inclusion criteria required patients to exhibit primary acute respiratory symptoms clinically relevant enough to necessitate hospital admission for monitoring at either the ward or ICU level, with or without the need for oxygen supplementation. In Angers, only patients in the highest severity stages were recruited. Standard care and clinical judgment by the physician on duty were uniformly applied to all included patients. The study did not involve randomization or blinding. A difference of at least 1.25 ng/mL in APL bloodstream content, observed in a pilot assessment, was considered “biologically significant”. Consequently, a targeted sample size of at least 50 patients per group was established to achieve an α of 0.05 and a power of 80%.

### 2.3. Defining Groups and Subsets: ARDS and SARS-CoV-2

All cohort patients admitted within 36 h for an acute and symptomatic respiratory condition were included. Daily identification, recruitment, consent, follow-up, and blood sample collection were managed by the Intensive Care Clinical Research Team. Patients were screened and tested for COVID-19 upon admission and categorized as either COVID+ or COVID− (control subjects were always COVID−). Subsets of patients were delineated based on severity: higher stages managed in ICU wards according to the Berlin 2012 definition for COVID− ARDS patients [40] and according to the same PaO_2_/FiO_2_ scale for COVID+ SARS patients (related to CoV-2) (https://www.cdc.gov/sars/index.html (Accessed on 30 September 2020), and lower stages managed on wards for respiratory symptomatic COVID− non-ARDS and COVID+ non-SARS inpatients. An expanded Berlin ARDS definition proposed in 2023 was applied for the use of high-flow nasal oxygen (HFNO) [41]. The severity criteria for ward assignment and subset allocation included O_2_ level > 6 L/min or the equivalent and/or the need for invasive mechanical ventilation (MV) at the ICU level; or O_2_ requirements ≤6 L/min with 40% FiO_2_ for SpO_2_ ≥ 90% on the floors and the need for non-invasive versus invasive mechanical ventilation, following COVID “MSSS” critical care committee recommendations. These criteria were also applied at the second center.

A small cohort of control subjects, matched for median age and sex to the patient groups, was recruited at the end of the study inclusion period. These control subjects were hospitalized for different medical conditions, but lacked acute respiratory symptoms and had a negative COVID-19 test within 24 h.

### 2.4. Patient Cohort Criteria

#### 2.4.1. Inclusion

- Age of 18 years or older;
- Admitted to ICU or ward ≤ 36 h prior;
- Confirmed or refuted active COVID-19 infection using a real-time reverse transcriptase–polymerase chain reaction (RT-PCR) test on a nasopharyngeal swab, intended for the qualitative detection of nucleic acids from SARS-CoV-2 (Roche Diagnostics, Montreal, QC, Canada) on the cobas^®^ 6800/8800 systems in Sherbrooke. Additionally, a real-time transcription-dedicated amplification assay (RT-TMA) was used for the Aptima™ SARS-CoV-2 assay on the Panther Instrument (Hologic) in Angers;
- Subset allocation based on symptoms and oxygen levels ±6 L/min or the equivalent and/or the need for invasive/non-invasive mechanical ventilation.

#### 2.4.2. Exclusion

- Age of under 18 years;
- Primary pulmonary embolism;
- Severe documented COPD or pulmonary fibrosis on home oxygen;
- Stages 3 and 4 lung cancer;
- Outside the inclusion time window;
- Moribund (end-of-life) patients or those requesting comfort care with a do-not-resuscitate order;
- Research team not available.

### 2.5. Time Points and Techniques for Data Collection

Data collection and blood sampling were performed every 7 days from baseline inclusion and continued up to 28 days, or until hospital discharge or patient death.

### 2.6. Demographic, Respiratory Physiological, Additive Therapy, and Outcome Data

Demographic information was recorded at the baseline. At the baseline and every 7 days thereafter, respiratory physiological data, including gas exchanges, oxygen supply needs and modes, ventilatory parameters, additional therapies (both pharmacological and non-pharmacological), and outcome data (including scores) were collected. These data were centralized in a computer system at both sites and transferred to a secure, encrypted, de-identified database named APEL-COVID (https://apel-COVID-ltb.cred.ca/) (accessed on 1 October 2020), specifically created for this study (the FRQS informatics platform) and accessible to both participating centers.

### 2.7. Biomarkers and Enzyme Activities

Two 10 mL blood samples (one in EDTA and one in heparin) were taken at baseline and every 7 days thereafter (if relevant). After rapid centrifugation at +4 °C, the plasma was aliquoted into dedicated 2 mL cryogenic polypropylene tubes with screw caps and stored frozen at −80 °C. EDTA plasma was used for biomarker measurements, and heparin plasma was used for enzymatic activity determinations.

#### 2.7.1. Biomarkers

Plasma obtained from EDTA-containing blood samples was centrifuged at 1600× *g* for 10 min. Apelin (APL) isoforms −36, −17, −16, −13, and −12, along with corresponding shorter degradation products, were measured using a commercially available ELISA kit (LifeSpan BioSciences, Lynnwood, WA, USA, cat. # LS-F25717, serial # 216589) with a detection range of 31.25–2000 pg/mL and a sensitivity of less than 18.75 pg/mL [42]. IL-6 plasma concentrations were measured with a commercially available ELISA kit (R & D Systems, Minneapolis, MN, USA, cat. # D6050, serial # P308610) with a detection range of 3.13–300 pg/mL, and a sensitivity of 0.7 pg/mL. SP-D plasma concentrations were measured using a commercially available ELISA kit (R & D Systems, cat. # DSFPD0, serial # P309989) with a detection range of 0.625–80 ng/mL and a sensitivity of 0.11 ng/mL.

#### 2.7.2. Enzyme Activities

- Neprilysin (NEP): 20 µL of plasma (from heparin-containing blood samples), 10 µL of substrate (5 mMol/L glutaryl-Ala-Ala-Phe-AMC; Peptides International, Louisville, KY, USA), and 50 µL of assay buffer (0.1 mol/L Tris-HCl, pH 7.6) were incubated at 37 °C for 30 min. The reaction was stopped by adding 10 µL of the NEP inhibitor phosphoramidon (0.1 mMol/L; Sigma, St. Louis, MO, USA) and incubating the samples on ice. Background controls were processed similarly, except that phosphoramidon was added before incubation at 37 °C. The samples were then incubated at 37 °C for 30 min with 10 µL of aminopeptidase M (500 mg/L, EMD Millipore, Burlington, MA, USA) and 5 mMol/L of EDTA. The reaction products were diluted in 3 mL of assay buffer, and fluorescence was measured at an excitation wavelength of 360 nm and an emission wavelength of 440 nm. NEP activity was calculated from the difference between the sample (S) and control (C) using the equation (S − C)/194 [42];
- Angiotensin converting enzyme 2 (ACE2): 2 µL of plasma (from heparin-containing blood samples) were incubated with a buffer (100 mM Tris-HCl, 600 mM NaCl, 0.5 mM ZnCl2, pH 7.5) and 20 µM of the quenched fluorescent substrate (Mca-Ala-Pro-Lys (Dnp)-OH; Enzo Life Sciences, Exeter, UK) at 37 °C for 16 h. Fluorescence was measured at 405 nm with excitation at 320 nm. The results were expressed as RFU/µL plasma/h [42];
- Lysyl oxidase (LOX): LOX activity in plasma (from heparin-containing blood samples) was quantified using a commercially available fluorometric assay kit (Abcam, Cambridge, UK, cat. # ab112139, serial # GR3197289-9) with a sensitivity of 40 ng/well. The fluorescence was detected at an excitation wavelength of 540 nm and an emission wavelength of 590 nm [21].

### 2.8. Ethics

Research Ethics Committee approvals were obtained for this study at both sites: Comité d’Éthique de la Recherche en Santé chez l’Humain du CIUSSS de l’Estrie-CHUS (#2020-3862) in Sherbrooke and Comité de Protection des Personnes (CPP DC 2016-2700), Biocollection cohorte maladies infectieuses (CRB-0118-FO-219-V01) in Angers. Due to the nature of the patient population, particularly those with acute respiratory failure, altered mental status, or major vital organ failure and shock, informed consent was often waived and initially obtained through a close friend or relative at both centers. Once capacity was regained, consent was sought directly from the patient, in accordance with the Mental Capacity Act in Canada (P-41 in Québec), when possible. This consent procedure was approved by both REC reviews in early November 2020.

### 2.9. Statistical Analyses

Categorical variables were expressed as frequencies (percentages). Continuous variables were presented as means (standard deviations) or as medians [interquartile ranges], depending on the distribution of the variables. Normality was assessed visually using histograms. Groups were compared using the chi-square test (or Fisher’s exact test where appropriate) and the Student’s *t*-test (or Mann–Whitney U test for non-normally distributed data). Missing data were reported as frequencies (percentages) in the tables. Listwise deletion was used for analysis, as missing data did not pose a significant issue. A significance level of 5% was considered, and results were obtained using GraphPad Prism 9.0 (version 9.3.1., GraphPad Software) and SPSS R v4.2.3.

OBJ 1: Biomarkers were compared between COVID− and COVID+ groups using multivariable linear regression. Biomarker outcomes were log-transformed. As this is a pre-post design, final values were considered for the outcome and adjusted for the baseline biomarker values. The variability of the endpoints between the groups was preliminarily assessed and found not to be differently distributed. Covariates included age, sex, and steroid use. The results were presented as adjusted fold changes (FC) with their 95% confidence intervals (95% C.I.). The model assumptions (normality and homoscedasticity) were validated with appropriate diagnostic plots.

OBJ 2: The strength of the association between baseline biomarkers and main outcomes was assessed using logistic regression (for death) or linear regression (for length of in-hospital stay and final SOFA score). The results were presented as odds ratios (OR) or mean differences (MD), respectively, along with their 95% confidence intervals. An interaction test was also conducted between the COVID− and COVID+ groups to assess possible synergistic effects.

## 3. Results

A total of 132 inpatients were included in this study between the end of October 2020 and mid-September 2021. This cohort included eleven control patients who were in-hospital for non-respiratory reasons.

### 3.1. Study Screening Allocation Algorithm

**Figure 1:**
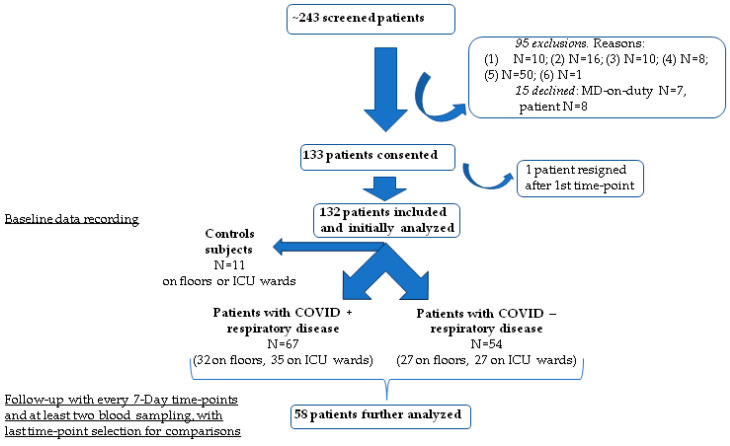
Study design: from screening to patient allocation. ARF: acute respiratory failure, ARDS: acute respiratory distress syndrome, SARS: severe acute respiratory syndrome.

This is shown in Figure 1. The primary aim of recruiting control subjects was to establish a matched standard for comparing biomarkers between groups and subsets.

### 3.2. General Characteristics of Studied Subjects

The general characteristics of the cohort and control subjects are displayed in Table 1. The distribution of these data in groups and subsets is shown in Table S1A,B, Supplementary. COVID− patients had higher APACHE II scores, but not higher SOFA scores compared to COVID+ patients (*p* = 0.0081, Supplementary Table S1A). Among the 27 COVID− ARDS patients, 25 (93%) exhibited direct injury (20 cases of pneumonia and 5 cases of aspiration), and the overall impairment of the parameters followed the trend of severity in the subsets (Supplementary Table S1B). Because blood collections were performed every 7 days after the baseline, 63 patients had only one biochemical measurement: 50 left before day 7, and 13 died before day 7.

Baseline comorbidities of cohort subjects are shown in Table 2.

### 3.3. First Descriptive Analysis

**Figure 2:**
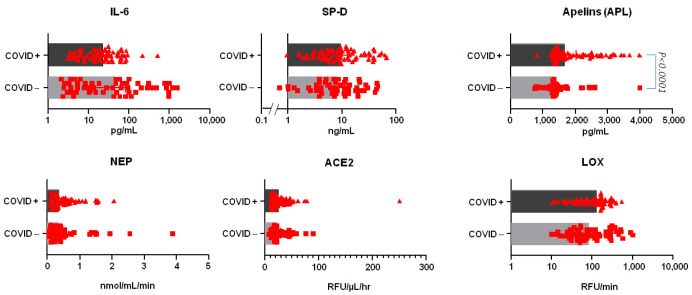
Baseline blood biomarkers in cohort patient groups. Data are displayed as scatter plots, with red triangles representing COVID+ patients and red squares representing COVID− patients. Bars indicate medians with interquartile ranges (IQR), with dark gray for COVID+ and light gray for COVID−. Data are shown on Log10 or linear scales on the x-axis. Biomarkers measured by EIA include IL-6, SP-D, and APL (upper panel), while enzyme bioactivities for NEP, ACE2, and LOX are shown in the lower panel. RFU: relative fluorescence units. Comparisons were made between COVID+ and COVID− patients. Data were first analyzed using one-way ANOVA with the Kruskal–Wallis test for non-parametric data, followed by Dunn’s multiple comparisons test. A significance threshold of *p* < 0.05 was used.

Baseline APL blood levels were higher in COVID+ patients compared to COVID− patients, while other biomarkers and enzyme activities did not show clear differences between the groups (Figure 2).

**Figure 3:**
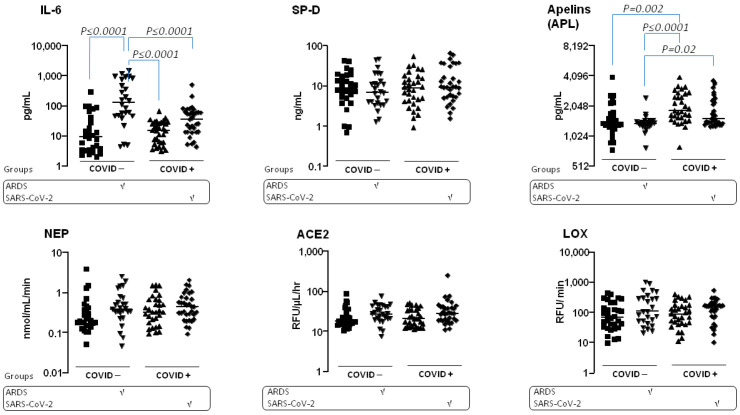
Baseline blood biomarkers in severity subsets of cohort patients. Data are displayed as scatter plots with medians and interquartile ranges (IQR) on Log10 or 2 scales on the x-axis. Biomarkers measured by EIA include IL-6, SP-D, and APL (upper panel), and enzyme bioactivities for NEP, ACE2, and LOX (lower panel). Four subsets are shown: severe COVID+ (SARS-CoV-2) in black lozenges; less severe COVID+ patients in black triangles; severe COVID− patients (ARDS) in inverted black triangles; and less severe COVID− patients in black squares. RFU: relative fluorescence units. Data were analyzed using one-way ANOVA with the Kruskal-Wallis test for non-parametric data or Fisher’s exact test, followed by Dunn’s multiple comparisons test. A significance threshold of *p* ≤ 0.05 was used.

Baseline APL values were elevated in all COVID+ severity subsets, whereas IL-6 levels were more increased in ARDS patients (Figure 3).

Comparisons of baseline biomarker data in between groups and subsets for cohort patients and control subjects are presented in Supplementary Figures S1 and S2.

**Figure 4:**
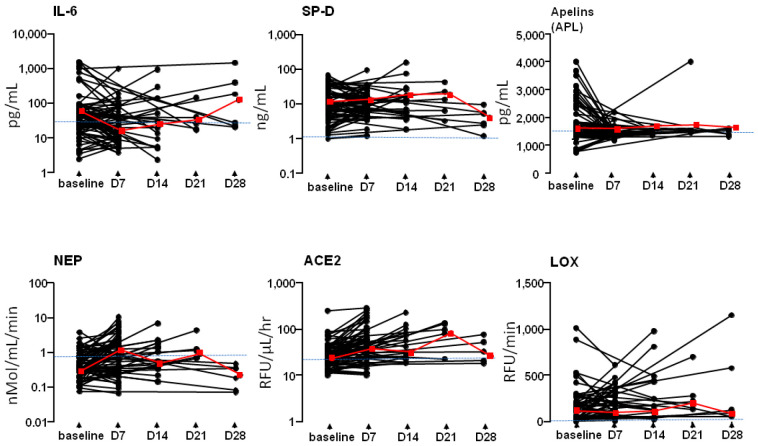
Time-course values of blood biomarkers in cohort patients. Data are displayed as individual black circle plots. Biomarkers were measured by EIA for IL-6, SP-D, and APL (upper panel) and enzyme bioactivity for NEP, ACE2, and LOX (lower panel). The median value for control subjects is indicated by a dashed blue line in each panel. Representative medians for cohort patients are shown with red lines and squares. Black circles representing individual values from the baseline to the respective endpoint are connected by black lines, illustrating variability (i.e., D7 to D28) among patients. All available data are presented.

The trends in the dynamic time course of blood biomarkers in the patient cohort are shown in Figure 4.

### 3.4. Subsequent Analyses

Subsequent analyses based on pre–post assessment for dynamic comparisons were performed on a cohort of 58 patients, with general characteristics displayed in Table 3.

The prevalence of corticosteroid use, higher APL values in the bloodstream, and the severity of the P/F ratio impairment were characteristic of COVID+ patients compared to COVID− patients. Regarding the selected biomarkers and enzyme activities, a linear regression model on baseline-adjusted parameters revealed that NEP degrading enzyme activity was increased in COVID+ patients (+43%, *p* = 0.029 vs. COVID− patients, Table 4A). This increase was more pronounced in SARS COVID+ patients (+65%, *p* = 0.032 vs. non-SARS COVID+ patients, and +73%, *p* = 0.005 vs. ARDS COVID− patients, Table 4B).

Additionally, enhanced LOX fibrosing activity was associated with death in this cohort (*p* = 0.037, Table 5A), and was particularly evident in SARS COVID+ patients (*p* = 0.05 vs. non-SARS COVID+ patients, Table 5B).

Finally, lower APL levels were trending towards being linked with a longer in-hospital stay (*p* = 0.055, Table 5A), and were notably associated with SARS COVID+ patients compared to ARDS COVID− patients (*p* = 0.048, Table 5B).

## 4. Discussion

This study aimed to compare two groups of patients with different etiologies of acute respiratory failure in relation to COVID status and two levels of severity. Biomarkers specifically targeting key pathophysiological events and potentially distinctive features were selected: three linked to inflammation, ACB leakage, and remodeling/fibrosis, and three others linked to the RAS and APL/APJ systems. At first glance, apart from APACHE II scores in the overall cohort, the clinical profiles of both COVID− and COVID+ patients were similar, including the severity of gas exchange impairment, the need for oxygen and ventilatory support, and survival rates. However, a subsequent and more detailed analysis of a more restrictive cohort with pre- and post-data revealed that COVID+ patients were more likely to receive corticosteroids and were more hypoxemic in this study.

Distinctive phenotypes were observed regarding biomarkers’ signatures and their association with outcomes. SP-D is the fourth recognized surfactant-associated protein, contributing to innate immunity in the lung, and is known for its value as a bloodstream marker of ACB impairment [15,16,17]. Hence, SP-D serves as a diagnostic marker for ACB leakage in ARDS and lung fibrosis, with elevated blood levels correlating with worse clinical outcomes [16,17,43]. In this cohort, although SP-D levels were equal at the baseline, the elevated SP-D was more strongly associated with COVID+ patients. A similar profile was recently described in SARS-CoV-2 patients in two studies [44,45], but not in another [46]. Despite being steroid-sensitive [47], SP-D levels remained high and were almost always linked to APACHE II scores in steroid-treated SARS-CoV-2 patients, suggesting that this treatment has a limited impact on ACB permeability.

IL-6 is an acute phase-response biomarker associated with detrimental outcomes during sepsis and ARDS [48,49]. With the COVID-19 pandemic, several neutralizing antibodies targeting IL-6 receptors were developed and trialed in over ten thousand SARS-CoV-2 patients to control the “cytokine storm,” which successfully reduced all-cause mortality [14]. Sustained elevated IL-6 levels in the bloodstream have been associated with a pro-inflammatory profile, disease severity, and mortality in recent studies on SARS-CoV-2 patients [50]. In this study, baseline IL-6 values were discriminative of severity in both patient groups but were not dynamically linked to any subsets or outcomes, although sustained high IL-6 levels have been associated with worse outcomes in SARS-CoV-2 patients treated with steroids [51,52].

The APL/APJ system, despite its high level of expression, is involved in lungs seriously affected by the SARS-CoV-2 virus at their epithelial–endothelial interface [24,25,26,27,28,29,52]. The cannabidiol- and melatonin-induced upregulation of APL expression has been shown to reduce ALI [28,53,54,55]. Furthermore, exogenous APL peptides (APL-13 and -36) have prevented endotoxin- and ventilation-induced ALI by reducing inflammation, acute ACB dysfunction, and TGFβ1-mediated endothelial-to-mesenchymal cell-related fibrosis [25,26,29,55,56,57,58,59]. In this study, as recently published by others [60], higher baseline APL plasma levels were associated with a shorter in-hospital stay. Specific degrading enzymes for APL peptides, such as ACE2 and NEP, have been described with steroid-driven upregulation/activation [61,62]. NEP, but not ACE2, was higher in COVID+ patients. Elevated NEP activity has been previously described in the bloodstream of ARDS patients [63], and this metalloproteinase has specific degrading activity on APL peptides, affecting their functionality [31]. Thus, higher potentially protective APL levels in COVID+ patients could be compromised by increased specific degrading pressure generated by NEP in the bloodstream.

Lung remodeling and fibrosing scars are emerging and prevalent healthcare issues in patients recovering from SARS-CoV-2, as highlighted by physiological and imaging reports [10,11]. LOX is a major enzyme orchestrating ECM proteins and collagen cross-linking and deposition in tissue and vital organs [64,65,66]. LOX blood levels and their variations have been proposed as tentative biomarkers of lung fibrosis activity at both the preclinical and clinical stages [20,67,68,69,70]. Higher LOX levels are associated with an increased risk of fibrosis progression and a greater risk of death [20]. It is also a regulator of lung vascular permeability [67]. Both LOX and the APL/APJ system are activated by Hypoxia-inducible Factor (HIF) [70,71,72,73,74]. LOX is a hypoxia-responsive gene [75], and upregulations of HIF and LOX are coordinated, especially in fibrogenesis processes [74,75,76,77]. The activation of LOX was very sensitive in this cohort and clearly associated with the risk of death in COVID+ patients, and this association was reinforced by severity. This strongest link observed in COVID+ patients is to be compared with the worrying prevalence (30–35% and more) of post-COVID interstitial lung disease with a fibrosis pattern [10,11]. Higher LOX levels were associated with increased SP-D leakage into the bloodstream due to ACB permeability/injury and with fibrosing events [77]. Mechanistically, TGFβ-driven maladaptive signaling to AEIIC has been recently reported as a driver of fibrosis after SARS-CoV-2 [78].

Of course, this study may lack the power to reinforce all the trends observed between biomarkers and outcomes in severity subsets. The second site recruited only patients within the highest severity subsets and the number of control patients should have been higher. Although, the selection of systemic biomarkers could always be debated. For these reasons and limitations, the generalization of these results should be approached with caution; however, APL remains an exploratory molecule with promising protective functions.

## 5. Conclusions

This study identifies a process-related biomarker signature and its evolution over time that delineates potentially useful differential trends between COVID+ and COVID− hospitalized patients. The APL/APJ and RAS systems are especially involved in COVID+ patients, with higher APL blood content associated with a shorter duration of hospital stay, rising NEP activity associated with severity, and LOX-associated sustained or increased remodeling/fibrosing activity linked with severity and increasing odds of death. Higher inflammation is consistently linked with poorer outcomes. Keeping apelin levels high in a controlled degradation environment seems to ensure a more favorable evolution with less complex lung repair. Thus, beyond the ‘cardiovascular box,’ the APL/APJ system shows promising potential for preventive and therapeutic approaches in acute lung injury (ALI) and sepsis, focusing on lung protection and rejuvenation [79,80].

## Figures and Tables

**Table 1:** Demographic, outcome scores, and physiological data. ND: not determined.

Population	Cohort Patients N = 121	Control Subjects N = 11
Age (years) Median [range]	67 [59–74]	61 [31–62]
Sex N (%)		
Male	74 (61.2)	6 (54.5)
Female	47 (38.8)	5 (45.5)
Group/Subset N (%) SARS (COVID+) Non-SARS (COVID+) ARDS (COVID−) Non-ARDS (COVID−)	35/121 (28.9%) 32/121 (26.4%) 27/121 (22.3%) 27/121 (22.3%)	11/11 (100%)
In-hospital deaths Days alive Median [range]	24/121 (19.8%) 9.5 [8.0–16.5]	0 (0%) ND
Survivors, length of in-hospital stay Median [range]	9 [5–14]	7 [3–11]
APACHE II score Median [range]	13 [8–19]	7 [5–13]
SOFA score, baseline Median [range]	3 [1–5]	0 [0–4]
PaO2/ FiO2 (P/F ratio), baseline Median [range]	232.5 [143.0–333.0]	ND
PEEP baseline (N = 35) Median [range]	10 [8–14]	ND

ND = Not determined.

**Table 2:** Chronic health conditions and comorbidities at the baseline in the cohort of patients hospitalized with respiratory symptoms. The most common comorbidities are displayed. Note: a single patient may have more than one comorbidity. COPD: chronic obstructive pulmonary disease.

Variable Comorbidities N (%)	COVID+ Patients N = 67	COVID− Patients N = 54
No	13 (19.4)	9 (16.7)
COPD	15 (22.4)	23 (42.6)
Hypertension	42 (62.7)	22 (40.7)
Heart Failure	13 (19.4)	15 (27.8)
Atherosclerotic Vascular Disease	7 (10.4)	6 (11.1)
Diabetes Mellitus	19 (28.4)	9 (16.7)
Chronic Kidney Failure	8 (11.9)	8 (14.8)
Chronic Liver Failure	0 (0)	2 (3.7)
Active Malignancy	7 (10.4)	8 (14.8)
Immunosuppression	3 (4.5)	4 (7.4)

**Table 3:** Demographic, baseline clinical and physiological variables and biomarkers and enzyme activities. All patients having a pre–post assessment and who are further included in logistic regression models are displayed. APACHE II score: acute physiological assessment and chronic health evaluation II score; SOFA score: sequential organ failure assessment score; SP-D: surfactant protein-D; IL-6: interleukin-6; APL: apelins; ACE2: angiotensin converting enzyme 2 activity; NEP: neprilysin activity; LOX: lysyl oxidase activity, P/F ratio: PaO_2_/FiO_2_ ratio, PEEP: positive end expiratory pressure, IQR: inter quartile range. Data were analyzed using one-way ANOVA with a Kruskal–Wallis test for non-parametric data. A significance threshold of *p* ≤ 0.05 was used. Significant *p*-values are shaded in grey.

Patients	All (N = 58)	COVID− (N = 27)	COVID+ (N = 31)	*p*-Value
Age—Mean (sd)	66.7 (12.7)	66.5 (14)	66.9 (11.6)	0.548
Female sex—N (%)	21 (36.2%)	12 (44.4%)	9 (29%)	0.223
Use of corticosteroids at baseline—N (%)	45 (77.6%)	16 (59.3%)	29 (93.5%)	0.002
Death—N (%)	10 (17.2%)	3 (11.1%)	7 (22.6%)	0.249
Length of In-Hospital stay (days)—Median [IQR]	14.5 [11–23]	15 [10.5–26]	14 [11.5–19.5]	0.778
APACHE II score—Median [IQR]	15 [10–21]	15 [9.5–23]	15 [10.5–20]	0.314
Baseline SOFA score—Median [IQR]	4 [2.25–7]	3 [2–8.5]	4 [3–6]	1.000
Baseline SP-D—Median [IQR]	9.57 [4.6–20.5]	7.93 [3.8–19.0]	9.65 [5.6–32.5]	0.244
Baseline IL-6—Median [IQR]	40.35 [14.7–83.8]	47.27 [11.9–98.8]	35.8 [18.8–63.8]	0.554
Baseline APL—Median [IQR]	1424 [1329–1826]	1371 [1309–1506]	1469 [1393–2223]	0.024
Baseline ACE2 activity—Median [IQR]	26.5 [17.4–41.2]	22.3 [17.4–42.9]	28.5 [18.5–38.8]	0.617
Baseline NEP activity—Median [IQR]	0.39 [0.21–0.97]	0.41 [0.19–1.3]	0.37 [0.3–0.8]	1.000
Baseline LOX activity—Median [IQR]	137.6 [44.2–218.2]	131.9 [55.4–293.5]	149.2 [37.3–192.9]	0.313
Baseline P/F ratio—Median [IQR]	144 [113–225.2]	193 [141.5–266]	130 [102–168]	0.006
Baseline PEEP—Median [IQR]	0 [0–10]	0 [0–10]	0 [0–10]	0.815

**Table 4:** Baseline adjusted biomarkers and enzyme activities and prognostic score and ventilatory physiological parameter differences between COVID+ and COVID− according to a linear regression model. (**A**) Group’s comparison with COVID− as the reference group, (**B**) severity subset’s comparisons with nonSARS COVID+; nonARDS COVID−; and ARDS COVID− as reference subsets, respectively. SOFA score: sequential organ failure assessment; SP-D: surfactant protein-D; IL-6: interleukin-6; APL: apelins; ACE2: angiotensin converting enzyme 2 activity; NEP: neprilysin activity; LOX: lysyl oxidase activity; P/F ratio: PaO_2_/FiO_2_ ratio; PEEP: positive end expiratory pressure; C.I.: confidence interval, IQR: Inter Quartile Range, FC: adjusted Fold-Change. Biomarkers and enzyme activities were log-transformed. All data were adjusted for age, sex, corticosteroids, and baseline values. Significant *p*-values are shaded in grey.

A	COVID− Median [IQR]				COVID+ Median [IQR]				FC	95% C.I.		*p*-Value
SOFA score	1 [0–3]				2 [1–4]				1.42	0.96	2.08	0.075
SP-D	5.67 [3.0–12.3]				17.17 [9.4–20.4]				1.63	0.98	2.69	0.058
IL-6	16.47 [6.5–77.5]				19.1 [8.5–36.6]				0.79	0.33	1.85	0.573
APL	1515 [1413–1580]				1446 [1400–1530]				1.00	0.91	1.10	0.981
ACE2	25.3 [19.4–57.5]				41.2 [26.4–82.6]				1.30	0.88	1.94	0.186
NEP	0.37 [0.1–0.8]				1.11 [0.4–1.9]				1.43	1.04	1.97	0.029
LOX	138.3 [101.2–391.8]				106.4 [73.8–253.2]				0.79	0.45	1.39	0.400
P/F ratio	357 [269–393]				223 [137–302]				0.65	0.49	0.88	0.005
PEEP	0 [0–0]				0 [0–8]				2.25	1.31	3.87	0.004
**B**	**nonSARS COVID+** **vs.** **SARS COVID+**				**nonARDS COVID** **−** **vs.** **ARDS COVID** **−**				**ARDS COVID−** **vs.** **SARS COVID+**			
	**FC**	**95% C.I.**		***p*-Value**	**FC**	**95% C.I.**		***p*-Value**	**FC**	**95% C.I.**		***p*-Value**
SP-D	1.26	0.59	2.67	0.532	1.41	0.73	2.72	0.294	1.42	0.72	2.81	0.302
NEP	1.65	1.05	2.60	0.032	0.97	0.54	1.74	0.921	1.73	1.19	2.50	0.005
P/F ratio	0.49	0.19	1.28	0.136	1.01	0.64	1.59	0.981	0.56	0.37	0.83	0.005
PEEP	2.61	0.80	8.58	0.108	0.81	0.47	1.40	0.434	2.83	1.29	6.24	0.011

**Table 5:** Strength of association between adjusted baseline biomarkers and enzyme activities and clinical outcome according to a linear regression model. (**A**) Group’s comparison with COVID− as the reference group, (**B**) severity subset’s comparisons with nonSARS COVID+; nonARDS COVID−; and ARDS COVID− as reference subset, respectively. SOFA score: sequential organ failure assessment; SP-D: surfactant protein-D; IL-6: interleukin-6; APL: apelins; ACE2: angiotensin converting enzyme 2 activity; NEP: neprilysin activity; LOX: lysyl oxidase activity; P/F ratio: PaO_2_/FiO_2_ ratio; PEEP: positive end expiratory pressure, C.I.: confidence interval, OR: Odds Ratio, MD: Mean Differences. Biomarkers and enzyme activities were log-transformed. All data were adjusted for age, sex, corticosteroids, and baseline values. Significant *p*-values are shaded in grey.

A Outcome Groups	-Death-					-Length of In-Hospital Stay-					-SOFA Score (Final)-				
	OR	95% C.I.		*p*-Value	Interaction Test	MD	95% C.I.		*p*-Value	Interaction Test	MD	95% C.I.		*p*-Value	Interaction Test
SP-D	1.03	0.99	1.1	0.134	0.722	0.1	−0.1	0.3	0.394	0.376	0.03	−0.01	0.08	0.176	0.605
IL-6	0.99	0.98	1.01	0.390	0.914	0.01	−0.002	0.02	0.114	0.944	−0.0003	−0.003	0.002	0.743	0.736
APL	0.99	0.99	1.01	0.915	0.361	−0.004	−0.01	0.0001	0.055	0.375	0.0001	−0.001	0.001	0.898	0.325
ACE2	1.02	0.99	1.05	0.127	0.704	0.004	−0.09	0.1	0.929	0.476	0.04	−0.02	0.02	0.723	0.717
NEP	0.89	0.31	1.30	0.708	0.831	0.53	−1.24	2.3	0.552	0.925	0.06	−0.35	0.47	0.774	0.622
LOX	1.001	1.0001	1.01	0.037	0.242	0.01	−0.01	0.02	0.361	0.811	0.002	−0.002	0.005	0.366	0.04
P/F ratio	0.99	0.98	1.001	0.221	0.961	−0.02	−0.05	0.01	0.128	0.467	−0.006	−0.01	−0.0004	0.037	0.321
PEEP	1.06	0.95	1.18	0.305	0.167	0.59	0.10	1.09	0.018	0.669	0.05	−0.06	0.17	0.361	0.289
**B** **Outcome** **Subsets**						**Death**									
						**OR**		**95% C.I.**				***p*-Value**		**Interaction Test**	
LOX-Only SARS (nonSARS COVID+ vs. SARS COVID+)						1.01		1.00		1.02		0.050		0.990	
LOX-Only ARDS (nonARDS COVID− vs. ARDS COVID−)						1.00		1.00		1.01		0.067		0.990	
LOX-Only Severe (ARDS COVID− vs. SARS COVID+)						1.00		1.00		1.01		0.167		0.290	
						**Length of In-Hospital Stay**									
APL-Only SRAS (nonSARS COVID+ vs. SARS COVID+)						0.00		−0.01		0.00		0.246		0.153	
APL-Only ARDS (nonARDS COVID− vs. ARDS COVID−)						−0.01		−0.02		0.00		0.192		0.287	
APL-Only Severe (ARDS COVID− vs. SARS COVID+)						−0.007		−0.013		−0.0001		0.048		0.247	

## Data Availability

The datasets used and analyzed during the current study are available from the corresponding authors on reasonable request.

## Supplementary Materials

The following supporting information can be downloaded at https://www.mdpi.com/article/10.3390/jcm13195919/s1, Figure S1: Baseline blood biomarkers in cohort patient groups and control subjects; Figure S2: Baseline blood biomarkers in severity subsets of cohort patients and control subjects. Table S1A: Demographic, outcome score, and physiological data. COVID+ and COVID− patients (groups); Table S1B: Demographic, outcome score, and physiological data for COVID+ non-SARS, SARS, COVID− non-ARDS, and ARDS (severity subsets).
