# Supplementary material for: Process-Specific Blood Biomarkers and Outcomes in COVID-19 Versus Non-COVID-19 ARDS (APEL–COVID Study): A Prospective, Observational Cohort Study
Source: J Clin Med. 2024 Oct 4;13(19):5919. doi: 10.3390/jcm13195919 (PMC11477790; doi:10.3390/jcm13195919)
Supplement: Supplementary file 1 [file jcm-13-05919-s001.zip › jcm-3229542-supplementary.pdf]

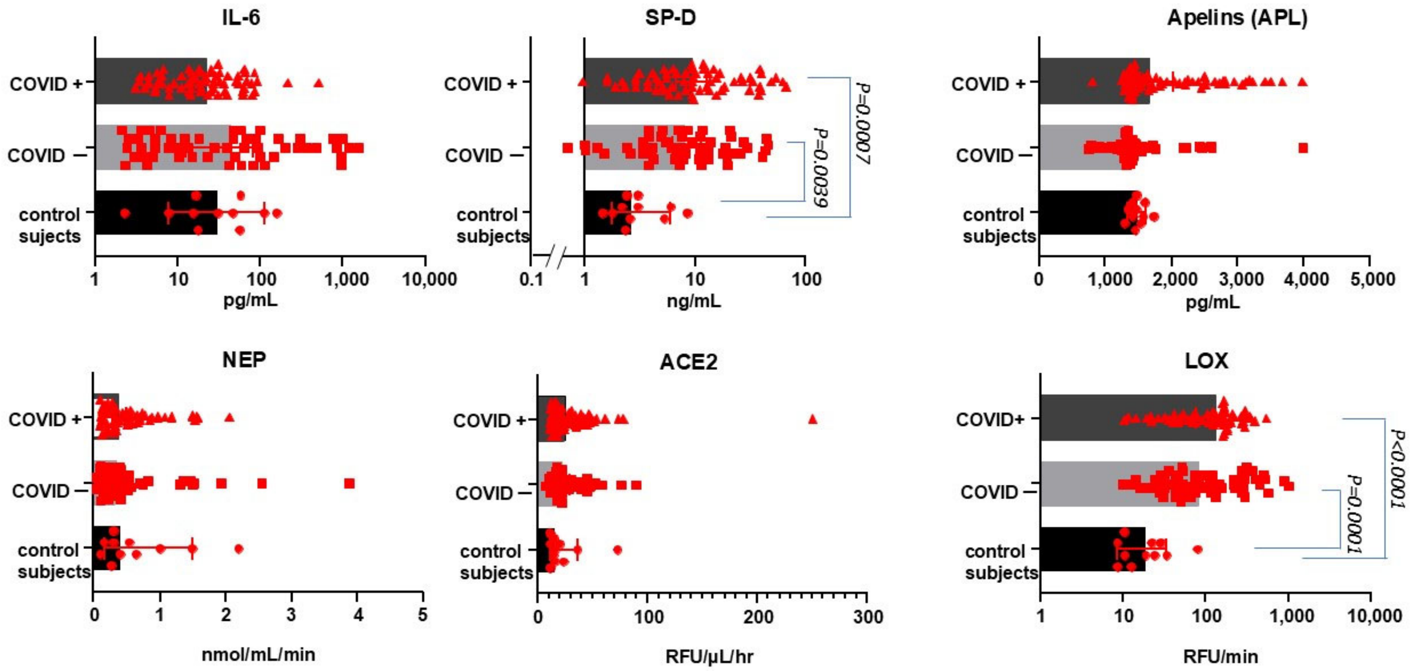

Figure 1 Supplement (Fig. S1): Baseline blood biomarkers in cohort patient groups and control subjects. Data are displayed as scatter plots, with red triangles representing COVID + patients, red squares representing COVID – patients, and red circles representing control subjects. Bars indicate medians with interquartile ranges (IQR), with dark gray for COVID + and light gray for COVID –. Biomarkers were measured by EIA for IL-6, SP-D, and APL (upper panel) and enzyme bioactivity for NEP, ACE2, and LOX (lower panel). Comparisons were made between COVID + patients, COVID – patients, and control subjects. RFU: relative fluorescence units. Data were analyzed using one-way ANOVA with Kruskal-Wallis test for non-parametric data, followed by Dunn's multiple comparisons test. A significance threshold of $P < 0.05$ was used.

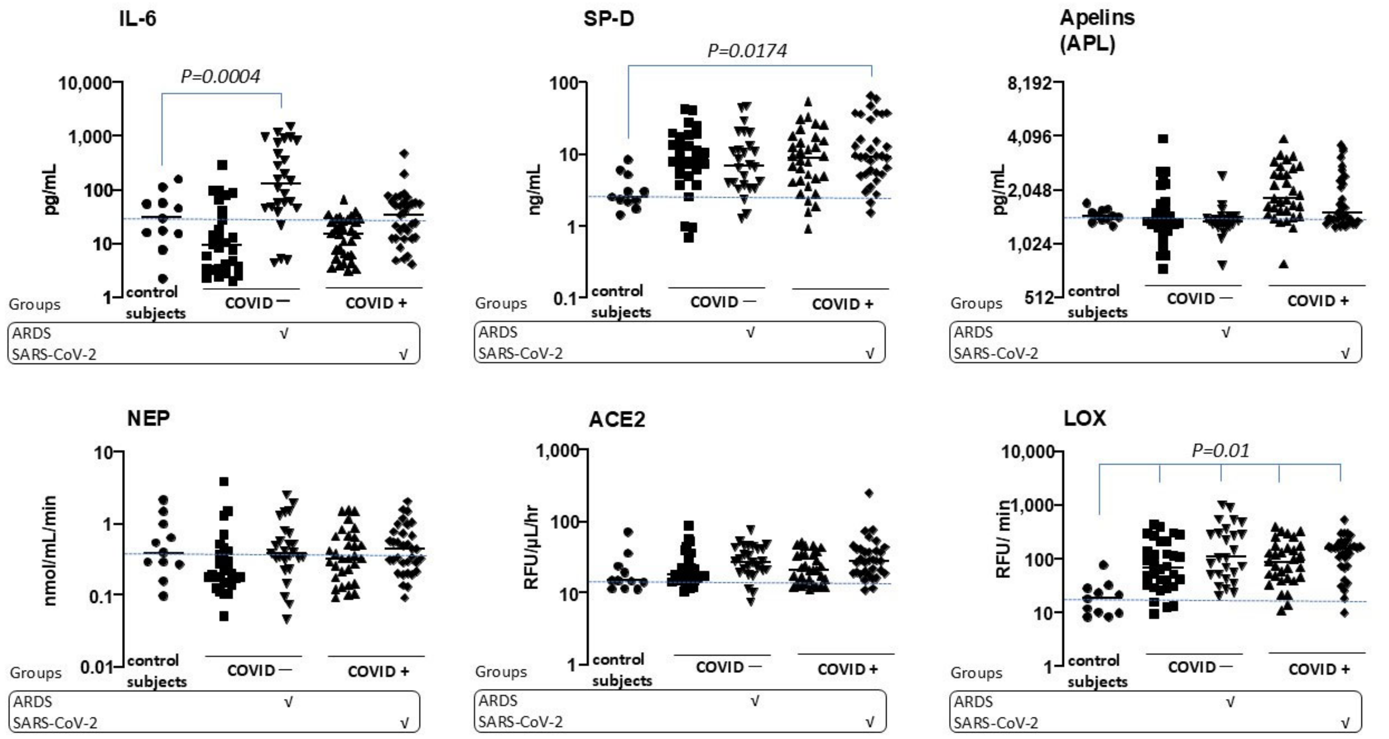

<u>Figure 2 Supplement (Fig. S2):</u> Baseline blood biomarkers in severity subsets of cohort patients and control subjects. Data are displayed as scatter plots with medians and interquartile ranges (IQR) on Log10 or 2 scales on the x-axis. Biomarkers were measured by EIA for IL-6, SP-D, and APL (upper panel) and enzyme bioactivity for NEP, ACE2, and LOX (lower panel). The figure includes three groups and five subsets: control subjects (black circles), severe COVID + (SARS-CoV-2) patients (black lozenges), less severe COVID + patients (black triangles), severe COVID – (ARDS) patients (inverted black triangles), and less severe COVID – patients (black squares). The median value for control subjects is indicated by a dashed blue line in each panel. RFU: relative fluorescence units. Data were analyzed using one-way ANOVA with Kruskal-Wallis test for non-parametric data or Fisher's exact test, followed by Dunn's multiple comparisons test. A significance threshold of P ≤ 0.05 was used.

Groups	COVID + patients N=67	COVID – patients N=54	*P-values*
Age (years)[a]	67 [61-73]	66 [59-75]	NS[b]
Sex n (%)<br>Male<br>Female	 44/67 (65.7%)<br>23/67 (34.3%)	 30/54 (55.6%)<br>24/54 (44.4%)	 NS[c]
In-hospital deaths n (%)<br><br>Days Alive[a]	12/67 (17.9%)<br><br>11.0 [9.7-16.5]	12/54 (22.2%)<br><br>9.0 [6.5-18]	NS[c]<br><br>NS[b]
Survivors, length of in-hospital stay[a]	8 [5-14]	9 [2-15]	NS[b]
APACHE II score[a]	12 [6-16]	14.5 [9.0-21.7]	*P=0.0081*[b]
SOFA score, baseline[a]	3.0 [1.0-4.5]	3.0 [1.2-7.0]	NS[b]
PaO_2/ FiO_2 (P/F ratio), baseline[a]	218.5 [130.8-308.8]	247 [158.5-381.0]	NS[b]
PEEP, baseline[a]	10 [9.5-14], n=16	9 [7-11.5], n=19	NS[b]
HFNO >30 liters/min[a]	19/67 (28.4%)	10/54 (18,5%)	NS[c]

Table 1A Supplement (Table S1A): Demographic, outcome score, and physiological data. COVID + and COVID – patients (groups). Selected parameters (a) are shown, and data are presented as the median [IQR] or proportions (%) and were compared using a one-way ANOVA with Kruskal–Wallis's test (b) or Fisher's exact test (c), with $P \leq 0.05$ as the threshold of significance. PEEP: Positive End Expiratory Pressure, HFNO: High-Flow Nasal Oxygen.

Subsets	COVID + patients		COVID – patients		
	**Non-SARS N=32**	**SARS N=35**	**Non-ARDS N=27**	**ARDS N=27**	***P-values***
Age (years)[a]	65 [58-72]	68 [64-73]	66 [59-74]	63 [59-78]	NS[b]
Sex n (%)					
Male	14/32 (43.75%)	25/35 (71.4%)	11/27 (40.7%)	19/27(70.4%)	*d,g: P< 0.03*[c]
Female	18/32 (56.25%)	10/35 (28.6%)	16/27 (59.3%)	8/27 (29.6%)	
In-hospital deaths n (%)	2/32 (6.25)	10/35 (28.6)	2/27 (7.4)	10/27 (37)	*d,f,g: P< 0.03*[c]
Days Alive[a]	16.5 [3-30]	14 [9-18.5]	6 [5-7]	14 [9-18.5]	*NS*[b]
Survivors, length of in-hospital stay[a]	6 [3-9]	12.5 [7-18]	7 [2-11]	16 [8-42]	*e,f,g,h: P< 0.04*[b]
APACHE II score[a]	6 [4.5-10]	15.5 [13-20]	10 [8-15]	21 [13-27]	*e,f,g,h: P< 0.04*[b]
SOFA score, baseline[a]	1.0 [0-2]	4 [3-6]	2 [1-3]	7 [4-9]	*e,f,g,h: P< 0.0004*[b]
PaO_2/ FiO_2 (P/F ratio), baseline[a]	257 [228-363]	122 [92-143]	286 [214-381]	157 [91-244]	*e,f g,h: P< 0.0001*[b]
PEEP, baseline[a]	0 [0-0], n= 2	0 [0-10], n= 16	0 [0-0], n= 3	7 [0-10], n= 19	*e,f,g,h: P< 0.03* [b]
HFNO >30 liters/min	n=0	n=19	n=0	n=10	*d,g: P< 0.002*[c]

<u>Table 1B Supplement (Table S1B):</u> Demographic, outcome score, and physiological data for COVID + non-SARS, SARS, COVID – non-ARDS, and ARDS (severity subsets). Selected parameters (a) are shown, with data presented as median [IQR] or proportions (%). Comparisons were made using one-way ANOVA with Kruskal-Wallis test (b) or Fisher's exact test (c), with a significance threshold of $P \leq 0.05$. Subset comparisons include: (d) non-ARDS vs. ARDS, (e) non-ARDS vs. SARS, (f) non-SARS vs. ARDS, (g) non-SARS vs. SARS, and (h) ARDS vs. SARS. PEEP: Positive End Expiratory Pressure, HFNO: High-Flow Nasal Oxygen.
